# Case Report: Fluzoparib for multiple lines of chemotherapy refractory in metastatic cutaneous squamous cell carcinoma with BRCA2 pathogenic mutation

**DOI:** 10.3389/fphar.2022.968060

**Published:** 2022-08-12

**Authors:** Xin Sun, Wenjuan Chen, Xiujuan Qu, Ying Chen

**Affiliations:** ^1^ Department of Medical Oncology, The First Hospital of China Medical University, Shenyang, China; ^2^ Key Laboratory of Anticancer Drugs and Biotherapy of Liaoning Province, The First Hospital of China Medical University, Shenyang, China; ^3^ Liaoning Province Clinical Research Center for Cancer, Shenyang, China; ^4^ Key Laboratory of Precision Diagnosis and Treatment of Gastrointestinal Tumors, Ministry of Education, The First Hospital of China Medical University, Shenyang, China

**Keywords:** cutaneous squamous cell carcinoma, PARPi, fluzoparib, BRCA2 germline mutation, case report

## Abstract

**Background:** Poly ADP-ribose polymerase inhibitors (PARPis) are widely used for patients with BRCA1/2 mutations. However, until now, there is no available evidence reported for the efficiency of PARPis in cutaneous squamous cell carcinoma (cSCC).

**Case presentation:** We presented a case of a 40-year-old man diagnosed with metastatic cSCC, relapsing after multiple lines of chemotherapy. Liquid biopsy detected a BRCA2 pathogenic germline mutation (c.3109C > T), indicating PARPis might be effective for this patient. The patient achieved tumor stability, and progression-free survival was five months without severe adverse effects after taking fluzoparib.

**Conclusion:** This result confirmed that PARPis were effective for metastatic cSCC patients with germline BRCA2 pathogenic mutations and provided a new treatment option for this group of patients.

## Introduction

The main types of skin cancers include basal cell carcinoma, squamous cell carcinoma, melanoma, and Merkel cell carcinoma (Garbe et al., 2022; Lebbe et al., 2015; Leiter et al., 2020). Among these, cutaneous squamous cell carcinoma (cSCC) accounts for 20% of nonmelanoma skin cancer cases and is the second most common type of skin cancer (Leiter et al., 2020). It may originate from the keratinocytes of the epidermis or its appendages and is a local invasion and likely to metastasize to other organs (Motley et al., 2002). Surgical excision is the treatment of choice for cSCC (Leiter et al., 2020). Ten-year survival after surgery exceeds 90% for cSCC but drops significantly when metastases occur (Varra et al., 2018). Chemotherapy, targeted therapy such as cetuximab, and immunotherapy have been adopted to treat cSCC patients, but the response rates of targeted therapy and chemotherapy were relatively low, and the duration of response was short (Weis et al., 2022).

Evidence from studies indicated that pathogenic or likely pathogenic BRCA1/2 germline mutations occurred in many cancers, including breast cancer (Borg et al., 2010; Kwong et al., 2012), ovarian cancer (Azribi et al., 2021), urothelial cancer (Carlo et al., 2020; Nassar et al., 2020), prostate cancer (Ghose et al., 2021), and so on. In prostate cancer, a study has shown that BRCA2 pathogenic mutations are connected with some clinicopathological parameters and indicated a poor prognosis (Ghose et al., 2021). In skin cancers, recent studies showed that BRCA1/2 mutations could be found in cutaneous melanoma (Johansson et al., 2019). Few studies reported BRCA1/2 mutations in cSCC. Thus, although poly ADP-ribose polymerase inhibitors (PARPis) have been successfully applied to treat ovarian cancer (Mirza et al., 2016), hereditary breast cancer (Han et al., 2018), and prostate cancer (Boussios et al., 2021b) with BRCA1/2 germline mutations, whether PARPis are effective in cSCC patients carrying BRCA1/2 germline mutations remains unclear, as no study has been conducted in cSCC.

In this case, we first reported the treatment of fluzoparib for metastatic cSCC patients with a BRCA2 pathogenic germline mutation and found remission to fluzoparib. This study first provided evidence for the efficiency of PARPis in treating metastatic cSCC.

## Case description

**FIGURE 1:**
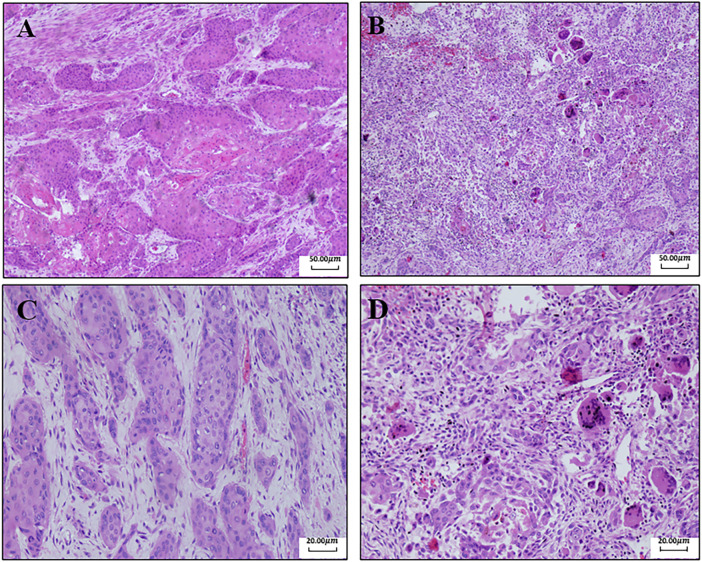
Pathological results of the right palm and right forearm. **(A)** Pathology of the right palm (40x): there are a lot of multinucleated giant cells and inflammatory cells in the tissue. Fibrosis occurs, and cells are swelling and deformed. These are the changes after chemotherapy. **(B)** Pathology of the right forearm (40x). **(C)** Pathology of the right palm (100x). **(D)** Pathology of the right forearm (100x).

**FIGURE 2:**
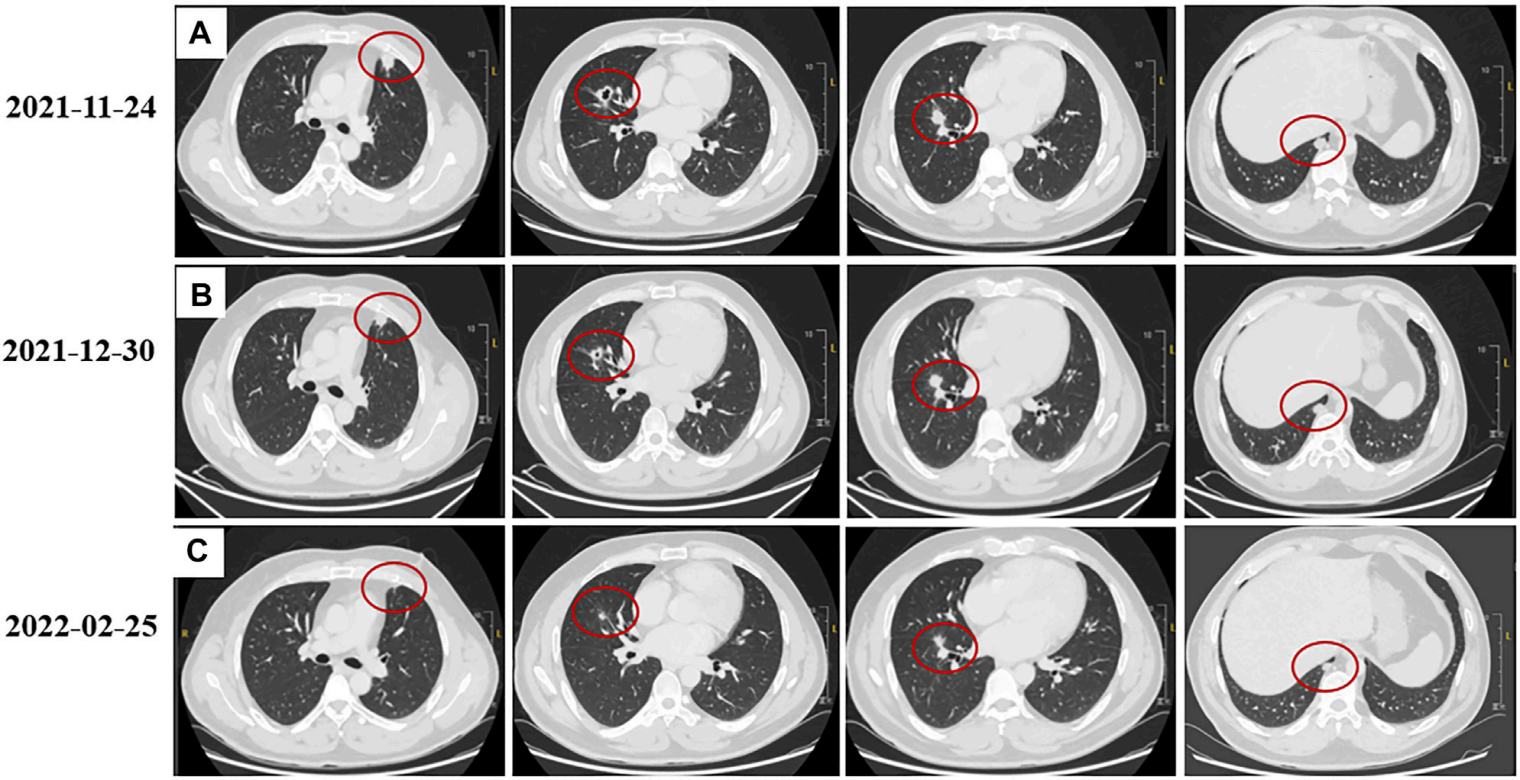
Tumor response to fluzoparib. **(A)**: Lung lesions before using fluzoparib. **(B)**: Lung lesions a month after using fluzoparib. **(C)**: Lung lesions three months after using fluzoparib. The red circles represent the target lesion.

**FIGURE 3:**
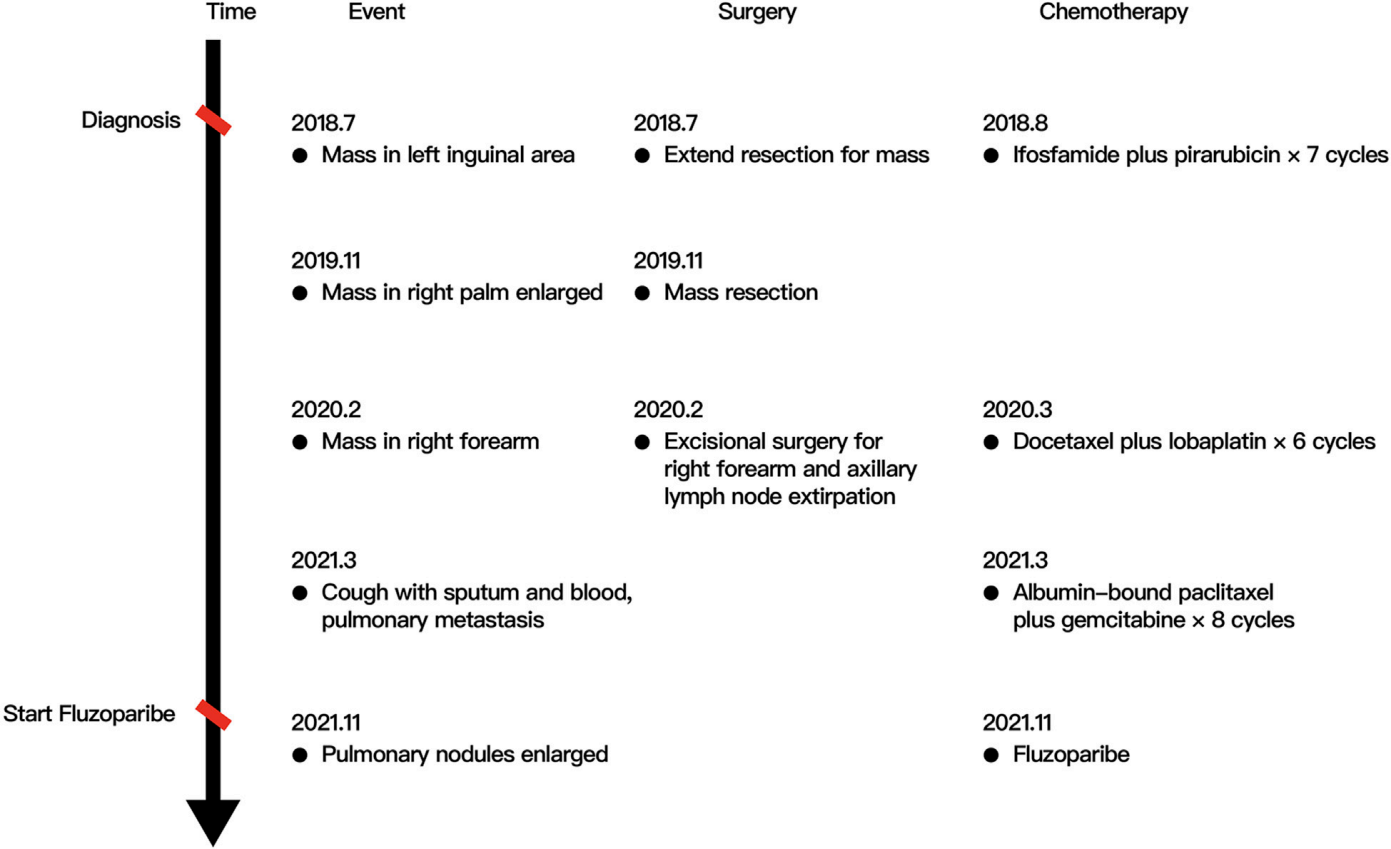
Case of a metastatic cutaneous squamous cell carcinoma patient.

A 40-year-old man first presented to the hospital with a mass in the left inguinal area in July 2018. It was about 2.0 cm * 3.0 cm, hard, and unpainful. The boundary of the mass was clear to some extent; however, the degree of the activity was poor. Extended resection was performed. The pathology demonstrated carcinosarcoma, which was mainly squamous cell carcinoma, and vascular tumor thrombus was positive. The patient denied any medical history and family history of cancer. The treatment after surgery was standard chemotherapy regimens combined with ifosfamide (2.5 g, every 21 days) with pirarubicin (30 mg, every 21 days) for seven cycles and combined cis-platinum (60 mg, every 21 days) with pirarubicin (30 mg, every 21 days) for the final cycle from August 2018 to May 2019. No adverse reaction occurred. In October 2018, the patient happened to find a mass in the palm of the right hand, which was shrunk during subsequent chemotherapy. However, in November 2019, the mass was suddenly enlarged to about 5.5 cm * 5.3 cm, painful, swollen, itchy, and accompanied by skin ulceration. The pathology after mass resection suggested well and moderately differentiated squamous cell carcinoma in the palm of the right hand, and it was difficult to identify the tumor tissue and normal tissue clearly. Immunohistochemistry (IHC) showed positive for P40, P63, and CK5/6; Ki67 was 50% positive. In February 2020, a mass appeared in the right forearm, and it was about 4.0 cm * 4.0 cm and ulcerated. After a series of examinations, excisional surgery for the right forearm and axillary lymph node extirpation was performed. The pathology suggested well and moderately differentiated squamous cell carcinoma, as found in relation to the mass in the palm. Furthermore, muscle tissue invasion, neural invasion, and lymph node metastasis (5/7) were also found upon pathological inspection. IHC showed positive for P40, P63, S-100, D2-40, CD34, and CK5/6; Ki67 was 40% positive. To get promising anti-tumor activity with less toxicity, postoperative chemotherapy on this occasion was combined with docetaxel (100 mg, every 21 days) and lobaplatin (50 mg, every 21 days) for six cycles from March 2020 to July 2020. During the treatment, grade 2 to 4 adverse effects were not observed. In January 2021, the patient began to cough with gradually increasing sputum and blood. Enhanced computed tomography (CT) in March 2021 revealed multiple pulmonary nodules in the right middle lobe and subpleural region of the left upper lobe (the largest one was about 1.9 cm), which were partially larger than those of a month before. The pathological consultation of the right palm and right forearm indicated squamous cell carcinoma (Figure 1). The patient was finally diagnosed with cSCC with inguinal area and pulmonary metastasis (stage IV) after a multi-disciplinary team discussion. Additional IHC of tumor in the left inguinal area showed negative expression of PD-L1 (combined positive score, CPS <1, by 22C3 pharmDx assay, Dako, Carpinteria, CA, United States), indicating immunotherapy was less effective for this patient. Then, the patient underwent a chemotherapy regimen consisting of albumin-bound paclitaxel (200 mg, d1/d8 of 3 weeks) and gemcitabine (2,000 mg, d1/d8 of 3 weeks) for eight cycles from March 2021 to September 2021. During treatment, enhanced CT showed significant tumor shrinkage of pulmonary nodules, and the best clinical efficacy reached partial remission. However, the patient presented grade 3 neutropenia, which was improved by pegylated recombinant human granulocyte colony-stimulating factor (PEG-rhG-CSF). In October 2021, enhanced CT implied disease progression. Her-2 was negative. Detection of BRCA1/2 germline mutations and other homologous recombination repair related genes for the patient in blood identified BRCA2 germline mutation (c.3109C > T) (Table 1), and all relative genes detected are listed in Supplementary Table S1. Considering there was no standard treatment for the patient, the patient was advised to take fluzoparib. The patient agreed and regularly took fluzoparib (150 mg orally twice a day) from November 2021. A month later, the patient achieved a stable state of tumor in enhanced CT. Three months later, pulmonary nodules were in regression again (Figure 2). Also, no severe adverse effects occurred. Tumor markers remained normal during the patient’s treatment. The patient has been progression-free for five months after fluzoparib treatment at the last follow-up in April 2022 (Figure 3).

**TABLE 1:** Detection result of BRCA1/2 germline mutations in a blood sample.

Tumor-specific mutations				
Gene	Nucleotide variation	Amino acid variation	Mutation type	Germline/somatic mutation
BRCA2(NM_000059.3)	c.3109C>T	p.(Gin 1037Ter)	Heterozygous mutation	Germline mutations

## Discussion

To our best knowledge, this case first proved successful treatment by PARPis in a patient with cSCC subjected to multi-line treatments.

One of the highlights in this case was that the initial symptom of the patient was atypical until the diagnosis of the cSCC in the right palm. It has been reported that approximately 3% of melanomas lack an identifiable primary, otherwise known as melanoma of unknown primary (Boussios et al., 2021a). However, for SCC, it usually shows a hard nodular keratinizing tumor or crusted tumor which could ulcerate. Sometimes, it could show as a non-keratinized ulcer. For this patient, he first showed a mass in the left inguinal area without any other symptoms. Examinations and pathology demonstrated SCC of the left inguinal area without other abnormalities. Considering that cancer may derive from either primary or secondary tumors, standard chemotherapy was used for the patient. During treatment, the development of cSCC in the right palm was observed, proving that cSCC might be the primary tumor for this patient and the mass in the left inguinal area arose from metastasis. A clear diagnosis provided evidence for subsequent therapy.

Another valuable finding in the case was that a pathogenic BRCA2 germline mutation (c.3109C>T) was observed in this patient, which is rarely reported in cSCC. This mutation occurs at 3109 bp of BRCA2, with a base “C″ changing to “T”, transforming glutamine to a stop codon. This mutation terminates the protein coding in advance, further affecting the degradation of mRNA and tumor development. Except for BRCA1/2, other genomic alterations related to homologous recombination deficiency have been recognized, including Fanconi anemia genes (BRIP1 and PALB2), the core RAD genes (RAD51C and RAD51D), and genes involved in homologous recombination pathways either directly (BARD1, NBN, and ATM) or indirectly (CDK12) (Ramus et al., 2015; Shah et al., 2022). Recent studies have reported that PAPRi therapy shows good efficiency in patients with homologous recombination deficiency (mainly BRCA1/2 mutations), and some targeted drugs have been approved by The United States Food and Drug Administration (FDA) to treat malignant cancers. For example, olaparib for treating ovarian and breast cancer was approved (Kim et al., 2015), and the III-phase NOVA trial showed niraparib could be used for ovarian patients with recurrence through treatment with platinum-based chemotherapy (Mirza et al., 2016). In high-grade ovarian cancer, the III-phase ARIEL3 trial showed patients who had ever responded to platinum-based chemotherapy could benefit from the maintenance of rucaparib (Coleman et al., 2017). In prostate cancer, olaparib was approved for metastatic castration-resistant prostate cancer patients with deleterious BRCA1/2 mutations with disease progression following androgen receptor signaling inhibitor treatment (Boussios et al., 2021b). PARPis for gastrointestinal cancers are also underway (Pilie et al., 2019). Some clinical studies have shown that PARPis could provide important benefits with acceptable toxicities when they were used for maintenance therapy after responding to platinum-based chemotherapy (Pilie et al., 2019). For this patient, he has suffered from multi-line treatments, but the disease was in progression. Expression of PD-L1 was low, suggesting he was unlikely to benefit from immunotherapy. BRCA2 was a pathologic mutation that was likely to benefit from PARPis. The patient exhibited platinum sensitivity because the metastasis occurred around six months after platinum-based chemotherapy. These suggest that the patient might benefit from PARPis in this scenario.

Fluzoparib is a type of novel PARPi on the basis of olaparib. It could inhibit the PARP1 enzyme and further could induce DNA double-strand breaks, G2/M arrest, and apoptosis in homologous recombination repair (HR)-deficient cells (Wang et al., 2019). It also showed good pharmacokinetics and stable anti-tumor activity, as well as a favorable toxicity profile for the treatment of many cancers (Wang et al., 2019; Li et al., 2022). A phase-II trial about ovarian cancer observed that patients with platinum-sensitive and BRCA1/2-mutant showed a 69.9% for objective response rate, The median follow-up duration was 15.9 months by 21 March 2020 (Lee, 2021; Li et al., 2021); fuzuloparib is also used to treat cancers of the pancreas, prostate, breast, and lungs in some phase-II or phase-III clinical trials (Lee, 2021). Moreover, fuzuloparib, used for treating ovarian cancer (including fallopian tube cancer or primary peritoneal cancer), has been approved in China. Although clinical trials by using PARPis in cSCC have not been conducted in China, the patient was willing to take an investigational therapy with strict informed consent, providing strong evidence for PARPi prescription.

Liquid biopsy of blood has been widely used to diagnose and treat lung cancer (Paweletz et al., 2016), gastric cancer (Shoda et al., 2017; Wu et al., 2006), breast cancer (Wallwiener et al., 2015; Mayor et al., 2017), and urothelial cancer (Agarwal et al., 2018). Studies also found that liquid biopsy could be used in oropharyngeal SCC (Haring et al., 2022) and cSCC (Haring et al., 2022). In this case, a liquid biopsy of blood detected the alterations in mutational status without invasion, providing evidence for the use of fluzoparib.

The strength of the case was that we presented a rare case in which the symptom of the patient was uncommon in cutaneous squamous cell carcinoma (cSCC). Moreover, we first showed a case using fluzoparib to treat cSCC patients with BRCA2 germline mutations, and the patient achieved a stable state. Our patient also thought he was benefiting from the regime in therapeutic effect, economy, and convenience. This might provide clues for similar patients. However, this is just a case report, and there are individual differences in response. The efficacy of PARPis in cSCC treatment remains to be confirmed by clinical trials.

In conclusion, our study first proved that cSCC patients, with BRCA2 pathogenic germline mutations and sensitivity to platinum-based chemotherapy, responded well to fluzoparib, suggesting that PARPis may be used for treating cSCC patients carrying BRCA1/2 germline or somatic mutations; detecting mutations is necessary for identifying the correct therapeutic regime.

## Data Availability

The original contributions presented in the study are included in the article/Supplementary Material; further inquiries can be directed to the corresponding author.
