# Supplementary material for: Case Report: Fluzoparib for multiple lines of chemotherapy refractory in metastatic cutaneous squamous cell carcinoma with BRCA2 pathogenic mutation
Source: Front Pharmacol. 2022 Aug 12;13:968060. doi: 10.3389/fphar.2022.968060 (PMC9411933; doi:10.3389/fphar.2022.968060)
Supplement: Supplementary file 1 [file Table1.docx]

**Supplementary table S1. Detection of BRCA1/2 germline mutations and other homologous recombination repair related genes for the patient**

| Gene Type | Gene |
| --- | --- |
| Homologous recombination repair related genes | BRCA1, BRCA2, ABRAXAS1, ATM, ATR, BAP1, BARD1, BPIP1, C11ORF30, CDK12, CHEK2, FANCC, FANCD2, FANCI, FANCL, MRE11, NBN, PALB2, PPP2R2A, PTEN, RAD50, RAD51B, RAD51C, RAD51D, RAD54B, RAD54L |
| Mismatch repair related genes | MLH1, MSH2, MSH6, PMS2, EPCAM |
| Other tumor related genes | CDH1, STK11, TP53 |
